# Design and characterisation of synthetic operons for biohydrogen technology

**DOI:** 10.1007/s00203-016-1322-5

**Published:** 2016-11-21

**Authors:** Ciaran M. Lamont, Frank Sargent

**Affiliations:** Division of Molecular Microbiology, School of Life Sciences, University of Dundee, MSI/WTB/JBC/DCTIR Research Complex, Dow Street, Dundee, DD1 5EH Scotland, UK

**Keywords:** Bacterial physiology, Hydrogen metabolism, Synthetic biology, Hydrogenase

## Abstract

Biohydrogen is produced by a number of microbial systems and the commonly used host bacterium *Escherichia coli* naturally produces hydrogen under fermentation conditions. One approach to engineering additional hydrogen production pathways is to introduce non-native hydrogenases into *E. coli*. An attractive candidate is the soluble [NiFe]-hydrogenase from *Ralstonia eutropha*, which has been shown to link NADH/NAD^+^ biochemistry directly to hydrogen metabolism, an activity that *E. coli* does not perform. In this work, three synthetic operons were designed that code for the soluble hydrogenase and two different enzyme maturase systems. Interestingly, using this system, the recombinant soluble hydrogenase was found to be assembled by the native *E. coli* [NiFe]-hydrogenase assembly machinery, and, vice versa, the synthetic maturase operons were able to complement *E. coli* mutants defective in hydrogenase biosynthesis. The heterologously expressed soluble hydrogenase was found to be active and was shown to produce biohydrogen in vivo.

## Introduction

Biohydrogen is biologically derived molecular hydrogen and is considered a possible replacement for fossil fuels (Benemann 1996). *Escherichia coli* produces hydrogen gas predominantly under fermentative conditions (Sargent 2016) using the formate hydrogenlyase complex that couples formate oxidation to hydrogen evolution (McDowall et al. 2014). One possible approach to engineering additional routes to hydrogen production in *E. coli* is the heterologous expression of non-native enzymes. An attractive proposition for engineering additional hydrogen metabolism into *E. coli* is to assemble functional NADH-dependent hydrogenases (Ghosh et al. 2013; Kelly et al. 2015), which would not compete directly with native sources of reductant for hydrogen production and potentially link the new hydrogenase activity directly into the anaerobic metabolism of the host organism. This is because when *E. coli* is cultured fermentatively a major metabolic challenge for the cell is the recycling of the NADH produced by glycolysis. This process is normally carried out, in the main, by the aldehyde/alcohol dehydrogenase AdhE.

The Knallgas bacterium *Ralstonia eutropha* (also called *Cupriavidus necator*) contains a soluble cytoplasmic enzyme that links NAD^+^/NADH directly to H_2_ biochemistry (Burgdorf et al. 2005a). The *R. eutropha* soluble hydrogenase (SH) is encoded by the *hoxFUYHWI* operon where HoxFU represent the diaphorase half of the enzyme; HoxYH is the [NiFe]-hydrogenase half; HoxI is a sixth subunit of the enzyme; and HoxW is a maturation protease required for final activation of the [NiFe]-hydrogenase catalytic subunit, HoxH (Burgdorf et al. 2005a). Biosynthesis of the Ni–Fe–CO–2CN^−^ cofactor located at the active site of a hydrogenase requires the activity of specific ‘maturase’ proteins (Böck et al. 2006). These include HypA and HypB (which are involved in nickel processing); the chaperone HypC; and HypD, HypE and HypF, which together build the Fe–CO–2CN^−^ half of the cofactor (Böck et al. 2006). In *R. eutropha* there are at least two versions of most maturase genes available, together with an extra accessory protein of less clearly defined function, HypX (Burgdorf et al. 2005a; Schiffels et al., 2013). Some reports suggest that the *R. eutropha* maturases are very specific for the assembly of their cognate [NiFe]-hydrogenases (Burgdorf et al. 2005a; Schiffels et al. 2013) and this is consistent with the hypothesis that different prokaryotic species require their own dedicated maturase machineries (Böck et al. 2006; Lubitz et al. 2014).

In this work, a synthetic biology approach was taken to producing an active *R. eutropha* soluble hydrogenase in an *E. coli* host. Synthetic operons encoding the SH and hydrogenase maturases were designed and characterised. An active SH was produced and hydrogen production was recorded.

## Materials and methods

### Strain and plasmid generation

Plasmids and bacterial strains used are listed in Tables 1 and 2, respectively. IC011 was constructed by modifying the IC010 strain (Deplanche et al. 2010) using a Δ*hybOA* allele present on the pMAK705 derivative pRAT58 and the method of Hamilton et al. (1989). HJ001 (as IC011, Δ*iscR*) and HJ002 (as IC011, Δ*iscR*, Δ*adhE*) were assembled by moving the pMAK705-based Δ*iscR* and Δ*adhE* alleles constructed by Kelly et al. (2015) sequentially into IC011 by homologous recombination (Hamilton et al. 1989). Synthetic operons were constructed in silico using the *OPTIMIZER* software (Puigbo et al. 2007). A ribosome binding site and linker (5′-AGGAGGAAAAAAAA-3′) was added before each synthetic gene (Kelly et al. 2015) and restriction sites placed at each ends of the gene. The final sequence was then synthesised by Biomatik Corp (USA) and sub-cloned into various expression vectors (Table 1).

**Table 1:** Plasmids used and constructed in this work

Plasmid name	Details	Antibiotic resistance	References
pUNI-PROM	As pT7.5 Tabor and Richardson (1985) with *tatA* promoter and RBS cloned EcoRI–BamHI	Amp	Jack et al. (2004)
pSU-PROM	As pSU40 Bartolome et al. (1991) with *tatA* promoter and RBS cloned EcoRI–BamHI	Kan	Jack et al. (2004)
pUNI-SH	As pUNI-PROM with synthetic *hoxFUYHWI* operon cloned BamHI–HindIII	Amp	This work
pUNI-A2-X	As pUNI-PROM with synthetic *hypA2B2F2C1D1E1X* operon cloned BamHI–HindIII	Amp	This work
pSU-A2-X	As pSU-PROM with synthetic *hypA2B2F2C1D1E1X* cloned BamHI–HindIII	Kan	This work
pUNI-A1-X	As pUNI-PROM with synthetic *hypA1B1F1C1D1E1X* cloned BamHI–HindIII	Amp	This work
pSU-A1-X	As pSU-PROM with synthetic *hypA1B1F1C1D1E1X* cloned BamHI–HindIII	Kan	This work
pQE80-SH	As pQE80 (Qiagen) with synthetic *hoxFUYHWI* operon cloned BamHI–HindIII	Amp	This work
pGP1-2	Encoding T7 polymerase under a temperature-dependent promoter	Kan	Tabor and Richardson (1985)

**Table 2:** *E. coli* strains used and constructed in this study

Strain	Relevant genotype	Antibiotic resistance	Source/references
MC4100	F^−^, *araD139*, Δ(*argF*-*lac*)U169, *ptsF25*, *deoC1*, *relA1*, *flbB5301*, *rspL150*	None	Casadaban and Cohen (1979)
IC010	As MC4100, ∆*hyaB*, ∆*hycE*	None	Deplanche et al. (2010)
IC011	As MC4100, ∆*hyaB*, ∆*hybOA*, ∆*hycE*	None	This work
HJ001	As MC4100, ∆*hyaB*, ∆*hybOA*, ∆*hycE*, ∆*iscR*	None	This work
HJ002	As HJ001, ∆*adhE*	None	This work
BEF314	As MC4100, ∆*hypB*-*E*::*cam*	Cam	Jacobi et al. (1992)
BW25113	F^−^, Δ(*araB*–*D*)567, Δ(*rhaD*–*B*)568, Δ*lacZ4787*(::*rrnB*-*3*), *hsdR514*, *rph*-*1*	None	Baba et al. (2006)
JW2696	As BW25113, Δ*hypA*::*kan*	Kan	Baba et al. (2006)
JW2697	As BW25113, Δ*hypB*::*kan*	Kan	Baba et al. (2006)
JW2698	As BW25113, Δ*hypC*::*kan*	Kan	Baba et al. (2006)
JW2699	As BW25113, Δ*hypD*::*kan*	Kan	Baba et al. (2006)
JW2700	As BW25113, Δ*hypE*::*kan*	Kan	Baba et al. (2006)
JW5433	As BW25113, Δ*hypF*::*kan*	Kan	Baba et al. (2006)
K38	HfrC, *phoA4*, *pit*-10, *tonA22*, *ompF627*, *relA1*, λ^+^	None	Lyons and Zinder (1972)

### ^35^S-methionine radiolabelling

Briefly, *E. coli* strain K38 [pGP1-2] was transformed with the vectors to be tested. Cultures were grown at 30 °C in M9 minimal medium (Sambrook and Russell 2001), containing all amino acids except methionine and cysteine, before transcription of T7 RNA polymerase was induced by heat shock at 42 °C for 15 min. Rifampicin was then added to inhibit the native RNA polymerase activity and a 1 ml aliquot was spiked with 0.01 μCi of ^35^S-Methionine. After incubating for 15 min, samples were heated to 105°C, separated by SDS-PAGE, fixed and visualised by autoradiography.

### Enzyme assays

In vitro hydrogen oxidation activity was measured spectrophotometrically using the H_2_-dependent reduction of methyl viologen (MV) in anaerobic cuvettes (Palmer et al. 2010). Strains were grown anaerobically at 37 °C without agitation for 16 h in 500 ml Duran bottles containing LB media supplemented with 0.4% (w/v) fumarate, 0.5% (v/v) glycerol and appropriate antibiotics. Cells were harvested by centrifugation at 2773×*g*, washed twice in 50 mM Tris.HCl pH 7.8, and the wet weights of cell pellets recorded. Pellets were then suspended in 3 ml 50 mM Tris.HCl pH 7.8 before lysis by French Press (8000 psi) and crude extracts prepared by centrifugation.

For in vivo hydrogen production assays, Hungate tubes containing 5 ml M9 media supplemented with 0.8% (w/v) glucose and 0.2% (w/v) casamino acids were inoculated with 50 μl of pre-culture. Tubes were then inverted and incubated without agitation at 37 °C for 72 h. Headspace hydrogen was quantified using a gas chromatograph (Shimadzu GC-2014). Nitrogen was used as the carrier gas with a flowrate of 25 ml min^−1^, and the total amount of hydrogen in the headspace (10 ml) was calculated based on a standard curve. Values were normalised by considering the relative optical density of the cultures and the culture volume.

## Results

### Synthetic *hox* and *hyp* operons are expressed in *E. coli*

**Fig. 1:**
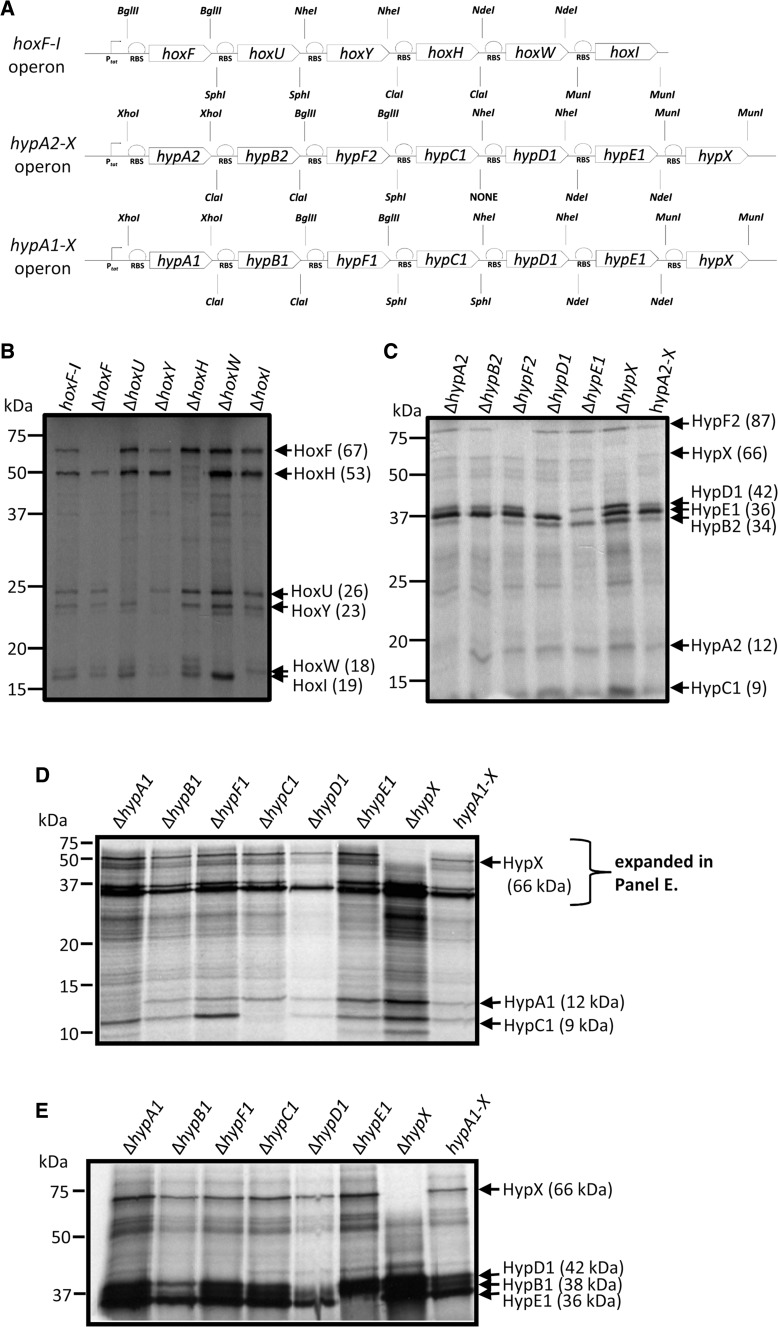
Synthetic *hox* and *hyp* operons for expression in *E. coli*. **a** Synthetic *hox* and *hyp* operons encoding the SH complex and associated maturases were designed for optimal expression in *E. coli*. The operons were initially cloned under the control of the constitutive *E. coli tat* promoter and the T7 promoter. Synthetic ribosome binding sites (RBS) were placed before each gene sequence, which had been previously codon optimised. Restriction sites bookended each synthetic gene to allow in-frame deletions to be constructed. ^35^S-Met radiolabelling was carried out in *E. coli* cells carrying either the synthetic *hoxFUYHWI* operon encoding the *R. eutropha* SH (**b**), the *hypA2B2F2C1D1E1X* operon (**c**) and the *hypA1B1F1C1D1E1X* operon (d) encoding *R. eutropha* maturase proteins. **e** The same samples as used in **d** were also subjected to SDS-PAGE on a 7.5% (w/v) polyacrylamide gel in order to get better resolution of protein bands ~40 kDa in relative molecular mass. Single gene deletions were prepared for each plasmid as indicated. Labelling of plasmid-encoded proteins was carried out in whole cells before samples were then separated by SDS-PAGE (12 or 7.5% w/v polyacrylamide), fixed, and visualised by autoradiography. Note that *hypC1* cannot be excised from its synthetic operon

To produce the soluble cytoplasmic [NiFe]-hydrogenase from *R. eutropha* in *E. coli* a synthetic operon was designed (Fig. 1a). The natural *hoxFUYHWI* gene order was maintained, but each synthetic gene was optimised for *E. coli* codon usage. Next, restriction sites were chosen to bookend each gene (Fig. 1a). The 5811 bp synthetic operon sequence was then synthesised and cloned into pUNI-PROM (Amp^R^), pSU-PROM (Kan^R^) and pQE-80 (Amp^R^) expression vectors (Table 1).

To test whether the *hoxFUYHWI* genes were being transcribed and translated in *E. coli*, the K-38[pGP1-2] strain was transformed with the pUNI-SH vector. This allowed T7 polymerase-dependent transcription of the operon and labelling of plasmid-encoded gene products with ^35^S-methionine. The radiolabelling experiments showed that pUNI-SH encodes at least six polypeptides (Fig. 1b). In order to assign each protein to a specific synthetic gene, a library of in-frame deletion plasmids was prepared. Following ^35^S-Met-labelling experiments, it is clear that the catalytic subunits of the SH HoxF and HoxH are correctly and stably synthesised. The remaining four polypeptides are also synthesised but, except for HoxY, appear to migrate in SDS-PAGE at slightly aberrant apparent molecular masses (Fig. 1b). However, the banding pattern on SDS-PAGE matches exactly that of previous work on the native (Burgdorf et al. 2005b) and recombinant (Schiffels et al. 2013) SH enzyme.

Activation of [NiFe]-hydrogenases requires specific maturases to build and insert the [NiFe] cofactor (Böck et al. 2006). The *R. eutropha* SH is reported to require the products of *hypA2*, *hypB2* and *hypF2* for activity (Wolf et al. 1998). In addition, *hypC1*, *hypD1*, *hypE1* and *hypX* all have roles to play (Böck et al. 2006; Burgdorf et al. 2005a; Schiffels and Selmer 2015). Thus, an 8213 bp synthetic *hypA2B2F2C1D1E1X* operon was assembled (Fig. 1a) and cloned into pUNI-PROM (Amp^R^) and pSU-PROM (Kan^R^) (Table 1). Construction of plasmid-borne in-frame deletions and in vivo pulse-labelling with ^35^S-methionine established that the maturation proteins were being produced in *E. coli* (Fig. 1c).

Finally, an alternative *R. eutropha* maturase operon, the 7115 bp *hypA1B1F1C1D1E1X* version, and derivatives were also constructed (Fig. 1a; Table 1). This synthetic operon was also found to be capable of producing protein product when examined by ^35^S-methionine labelling (Fig. 1d). Many of the encoded proteins can be readily identified; however, HypF1 (predicted to be 40 kDa) is not clear (Fig. 1d). A follow-up SDS-PAGE experiment was designed to generate greater separation in the 40 kDa region (Fig. 1e). In this case, HypB1, HypD1 and HypE1 could be readily identified; however, the synthesis of HypF1 could not be established (Fig. 1e).

### The synthetic maturase operons are functional and can activate *E. coli* [NiFe]-hydrogenases

**Fig. 2:**
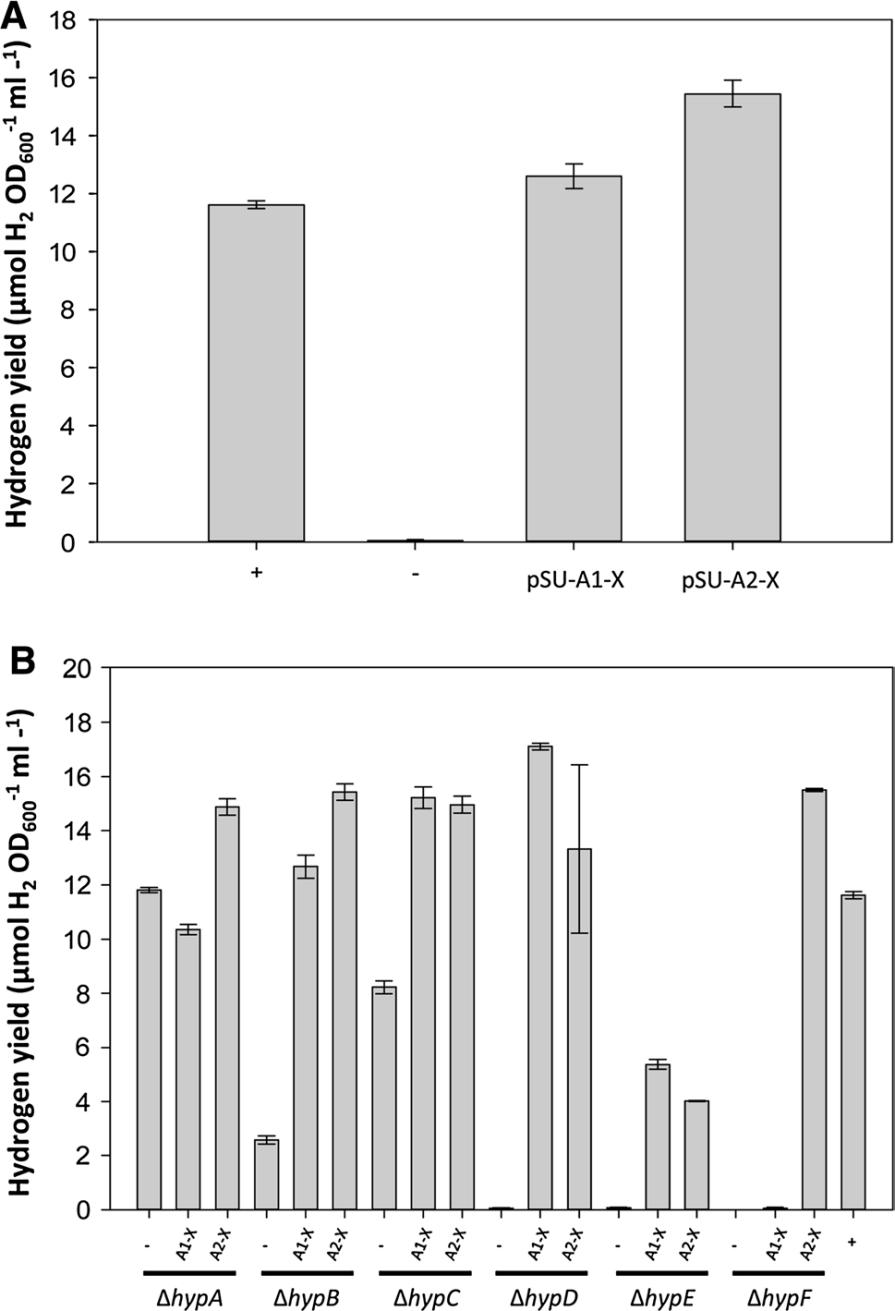
The synthetic maturase operons can complement *E. coli hyp* mutants. **a** *E. coli* strain BEF314 (Δ*hypBCDE*) was transformed with a pSU-PROM vector control (−) or vectors encoding the *hypA1*-*X* synthetic operon (pSUA1-X) or synthetic *hypA2*-*X* operon (pSUA2-X). **b** The Keio collection version of single *E. coli* deletion mutants in each of *hypA*, *hypB*, *hypC*, *hypD*, *hypE* and *hypF* were transformed with either empty vector (−) or pUNI-PROM-based vectors encoding the *hypA1*-*X* synthetic operon (A1-X) or synthetic *hypA2*-*X* operon (A2-X). In all cases, strains were grown fermentatively in M9 minimal media supplemented with 0.8% (w/v) glucose before gas chromatography was used to quantify any H_2_ accumulated in the headspace after 48 h of incubation at 37 °C. The positive control (+) is the *E. coli* strain MC4100. *Error bars* represent SEM (*n* = 3)

To test functionality of the maturase operons, genetic complementation of *E. coli* mutant strains compromised in [NiFe] cofactor biosynthesis was undertaken. The *E. coli* BEF314 strain (Δ*hypBCDE*) is devoid of hydrogenase activity (Jacobi et al. 1992). When BEF314 (Δ*hypB*-*E*) was transformed with plasmids encoding HypA1-X and HypA2-X, fermentative hydrogen production was restored to native levels (Fig. 2a), indicating that the *E. coli* FHL had been activated.

Next, individual mutants in each of the *hypA*-*E* and *hypF* genes were examined (Fig. 2b), beginning with *hypA*. As previously reported (Jacobi et al. 1992), the *hypA* mutant retains some hydrogenase activity (Fig. 2b). Indeed, under the growth conditions and assay type chosen, here the *hypA* mutant was able to accumulate hydrogen to the same level as the control strains (Fig. 2b).

HypB is a nickel-binding NTPase (Böck et al. 2006), and fermentative H_2_ production by the *E. coli hypB* mutant is low (Fig. 2b). This phenotype can be rescued by production of either HypA1-X or HypA2-X (Fig. 2b), and this demonstrates that both HypB1 and HypB2 are functional.

Next, a Δ*hypC* strain was tested. HypC is a small protein that acts as a bridge between the initial [NiFe] cofactor assembly proteins and the empty apoenzyme (Böck et al. 2006). In the hydrogen accumulation assay employed here, the Δ*hypC* mutant is able to demonstrate clear hydrogen production activity (Fig. 2b). Nevertheless, co-expression of plasmids encoding HypA1-X and HypA2-X returned hydrogen evolution to native levels (Fig. 2b), suggesting that *R. eutropha* HypC1 can substitute for *E. coli* HypC.

The operons encoding HypA1-X and HypA2-X can also rescue the hydrogenase-null phenotype of a *hypD* mutant (Fig. 2b). However, the complementation of *E. coli hypE* and *hypF* mutants, which encode two proteins that interact extensively (Stripp et al. 2015), is less compelling (Fig. 2b). The *R. eutropha* HypE1 protein (which is identically encoded in both *hypA1*-*X* and *hypA2*-*X* plasmids) is able to complement a Δ*hypE* mutant, although hydrogenase activity is clearly reduced (Fig. 2b). However, in the absence of endogenous *E. coli hypF*, it seems that *R. eutropha hypF1* is completely inactive in this assay (Fig. 2b).

### Production of an active SH in *E. coli*

To explore the activity of the SH, an *E. coli* host strain (HJ002) was prepared that lacked key native hydrogenase genes (Table 2) and also carried an Δ*iscR* allele, which can be beneficial for heterologous production of metalloenzymes (Kelly et al. 2015), and a Δ*adhE* allele, with a view to increasing the availability of the NADH substrate under fermentative conditions (Ghosh et al. 2013).

**Fig. 3:**
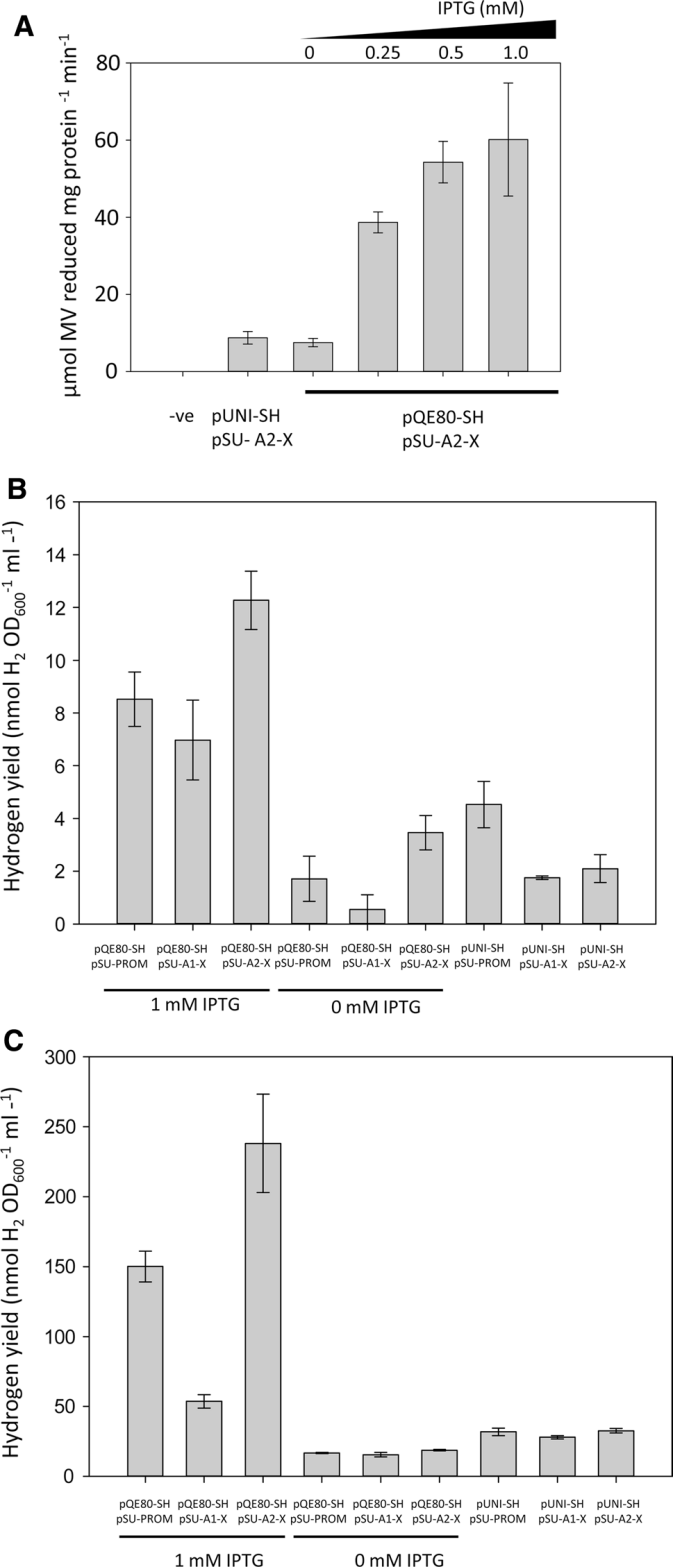
An active SH in *E. coli*. **a** *E. coli* host strain HJ002 (Δ*hyaB*, Δ*hybOA*, Δ*hycE*, Δ*iscR*, Δ*adhE*) was transformed with pUNI-PROM/pSU-PROM (−ve control); pUNI-SH/pSU-A2-X, or pQE80-SH/pSU-A2-X. Transformants were used to inoculate 500 ml LB media supplemented with 0.5% (v/v) glycerol, 0.4% (w/v) fumarate and appropriate antibiotics. The pQE80-SH containing cultures were additionally supplemented with 0, 0.25, 0.5 or 1 mM IPTG where indicated. Cultures were incubated at 37 °C overnight without shaking to induce anaerobiosis. Crude cell extracts were prepared and assayed for hydrogen-dependent reduction of methyl viologen. **b** *E. coli* strain HJ002 was transformed with either pQE80-SH or pUNI-SH together with either a control plasmid (pSU-PROM) or a plasmid carrying the synthetic *hypA1*-*X* operon or the *hypA2*-*X* operon. Transformants were used to inoculate sealed Hungate tubes containing 5 mL M9 media supplemented with 0.8% (w/v) glucose, 0.2% (w/v) casacids and appropriate antibiotics. A single end-point GC assay was used to determine the yield of hydrogen following ~72 h of incubation. *Error bars* represent SEM (*n* = 3). **c** Bacterial growth and gas chromatography was carried out as in **b**; however, 100 µM FeCl_3_ (final concentration) was added to the cultures from the outset. *Error bars* represent SEM (*n* = 3). Cultures were prepared under stringent anaerobic conditions where the media was sparged for 1.5 h with N_2_; then, in an anaerobic cabinet, Hungate tubes were filled with 5 mL of the sparged media, sealed, and finally inoculated with the strains to be tested

The HJ002 (Δ*hyaB*, Δ*hybOA*, Δ*hycE*, Δ*iscR*, Δ*adhE*) strain was transformed with a plasmid encoding *hypA2*-*X* operon and then co-transformed with pQE80-SH or pUNI-SH. The pQE80-SH vector, when uninduced, resulted in a comparable level of MV-linked hydrogenase activity to that of the pUNI-SH (Fig. 3a). Induction with progressively higher concentrations of IPTG correlated with a corresponding increase in hydrogenase activity (Fig. 3a). This assay demonstrates that the [NiFe]-hydrogenase half of the SH has been assembled and activated.

Next, the ability of the SH to produce H_2_ in vivo was assessed. The HJ002 (Δ*hyaB*, Δ*hybOA*, Δ*hycE*, Δ*iscR*, Δ*adhE*) strain was transformed with either pQE80-SH or pUNI-SH together with either a control plasmid (pSU-PROM) or a plasmid encoding *hypA1*-*X* or *hypA2*-*X*. Hydrogen gas in the headspace was determined by gas chromatography in a single end-point assay (Fig. 3b). The data show that there is H_2_ production from strain HJ002 carrying pQE80-SH, even without inclusion of the synthetic maturases (Fig. 3b). The native *E. coli* maturases are clearly able to assemble an active SH (Fig. 3b), thus partly corroborating the reciprocal experiment where the *R. eutropha* maturases were able to activate *E. coli* hydrogenase activity (Fig. 2). Furthermore, despite the IPTG-induced pQE80-SH strain having a similar level of MV-linked hydrogenase activity to the pUNI-SH strain (Fig. 3a), the ability to evolve H_2_ in vivo was greatly improved by using the pQE80-SH system (Fig. 3b).

During the course of this work, two further studies reported the reconstitution of SH activity in an *E. coli* host (Ghosh et al. 2013; Schiffels et al. 2013). In both cases, the addition of 100 µM iron salts to the growth medium was required for maximal activation of the hydrogenase (Ghosh et al. 2013; Schiffels et al. 2013). Thus, the synthetic operons under investigation here were also tested in growth media containing 100 µM FeCl_3_ (Fig. 3c). In this case, maximum levels of H_2_ evolution were recorded at 250 nmol H_2_ ml^−1^ OD^−1^ (Fig. 3c) compared to 12 nmol H_2_ ml^−1^ OD^−1^ without iron supplementation (Fig. 3b).

## Discussion

### Production of an active *R. eutropha* SH in *E. coli*

The *R. eutropha* soluble [NiFe]-hydrogenase enzyme is an attractive system to biotechnologists for several reasons. The SH enzyme is not membrane-bound, like so many other hydrogenases, and this lends the possibility to generate ‘cell factories’ producing vast quantities of active enzyme in the cell cytoplasm. In addition, the ability to link hydrogen oxidation to NAD(P)H cofactor regeneration means this enzyme could have several industrial applications working together with other NAD(P)H-dependent biochemical reactions. Finally, the natural oxygen tolerance of this enzyme (Burgdorf et al. 2005a) may give it an advantage as a possible H_2_ producer over the fragile O_2_-sensitive [FeFe]-hydrogenase class.

Recent advances have been reported in the production of the SH in *E. coli* B strains and *E. coli* K-12 strains. Both (Schiffels et al. 2013) and Ghosh et al. (2013) worked with the native *R. eutropha* sequences, rather than synthetic sequences as described here, and had differing overall aims to their studies.

Schiffels et al. (2013) aimed to develop an overexpression system to allow purification of the recombinant enzyme. To this end, a plasmid-based system was constructed using the native *R. eutropha* genes for the SH and associated maturases to allow high-level production and purification of an active SH in *E. coli* BL21(DE3), a normally hydrogenase deficient strain (Pinske et al. 2011). In this case, increasing extracellular nickel salts (with levels above 1 µM being inhibitory), or providing an extra nickel transporter, proved useful in increasing yield of active enzyme for purification (Schiffels et al. 2013). Interestingly, Schiffels et al. (2013) found that production of an active SH was dependent on co-expression with the *R. eutropha hypA2*, *hypB2*, *hypC1*, *hypD1*, *hypE1*, and *hypF2* genes. This is in contrast to the data described here, where the *E. coli* maturases could active the SH (Fig. 3). One difference between the two studies could be that the SH expression levels induced by Schiffels et al. (2013) were likely orders of magnitude greater than achieved here. Alternatively, however, it should be noted that the BL21(DE3) strain used by Schiffels et al. (2013) may well be defective in the global regulator FNR, which is necessary for full expression of nickel uptake and *hypBCDE* in *E. coli* (Pinske et al. 2011).

The work of Ghosh et al. (2013) had a similar aim to that described here—integration of an NADH-dependent [NiFe]-hydrogenase into *E. coli* metabolism. Ghosh et al. (2013) cloned the native SH genes behind a strong promoter and demonstrated very high (2 mol H_2_ per mole glucose) hydrogen gas production levels in an *E. coli* K-12 strain that had been deleted for native hydrogenase genes. Interestingly, in this case, the SH was able to substitute for the main alcohol dehydrogenase (AdhE) and restore growth to an *adhE* mutant under fermentative conditions (Ghosh et al. 2013). Moreover, this growth effect was dependent on supplying 25 µM nickel salts to the medium (Ghosh et al. 2013). The synthetic system described in this work was unable to restore fermentative growth to an *E. coli adhE* mutant on minimal media, and even the native *E. coli* K-12 control strains were unable to reach 2 mol H_2_ per mole glucose in this experimental setup. Clearly, expression levels must be very different between the Ghosh et al. (2013) experiments and those described in this work.

The work presented here used *E. coli* K-12 as a host strain and took an alternative synthetic approach to the design of the expression system. All gene sequences were designed by back-translation from protein products and arranged in synthetic operons under the control of single promoters. The modular design of the operons allows facile removal and replacement of each coding sequence, which could allow for future directed evolution or further biochemical studies. This system was found to successfully produce each individual subunit of the SH, and an active [NiFe]-hydrogenase was assembled that was capable of H_2_ evolution in vivo. In this system, higher level expression from the T5 promoter (pQE-80-derived) was needed to induce the highest hydrogen production levels.

### On the role of maturases in the biosynthesis of [NiFe]-hydrogenases

One relatively surprising result reported here is that active *R. eutropha* SH could be assayed in *E. coli* in the absence of its dedicated accessory proteins, thus demonstrating that, in this system, the *E. coli* maturases could recognise and activate HoxH. This is interesting because the latter stages of cofactor insertion require some degree of protein–protein interactions, in particular when a HypC-type protein makes direct contact with the catalytic subunit. In the case of *E. coli* [NiFe]-hydrogenase-3, the HypC protein forms a stable complex with HycE until the cofactor loading is complete (Drapal and Böck 1998). *E. coli* has a second HypC homologue, HybG that can substitute when HypC is missing (Blokesch et al. 2001), and it seems also that these proteins can contribute to maturation of the SH. Indeed, the HypC protein from *Dehalococcoides mccartyi* was also recently shown to be able to activate the *E. coli* [NiFe]-hydrogenases (Hartwig et al. 2015).

It was pleasing that the converse experiment also upheld the hypothesis of cross-talk between the different maturation systems. There, the *E. coli* hydrogenases could be activated by the synthetic maturase operons designed here. This was very important in showing that the maturases were functional. The exception was *R. eutropha* HypF1, which is an unusually truncated version of the protein, which was found to be inactive. HypF2, which is normally co-produced with the SH itself, more closely resembles the native *E. coli* protein (Wolf et al. 1998). Note, however, that production of HypF1 was also not clear in the ^35^S-Met-labelling experiment (Fig. 1) and taken altogether these data show that the *hypA1*-*X* operon may not be producing an active HypF1 protein. Overall, however, these complementation experiments suggest some previously unsuspected promiscuity may exist between cofactor insertion proteins from different biological systems.

## Concluding remarks

In this work, a synthetic biology approach has been taken to engineering additional hydrogenase biochemistry into *E. coli*. A functional *R. eutropha* SH was produced and, somewhat surprisingly, could be activated by the native maturases found in the host strain. Future work will concentrate on optimising this synthetic system and engineering further non-native hydrogenases.

## References

[CR1] Baba T, Ara T, Hasegawa M, Takai Y, Okumura Y, Baba M, Datsenko KA, Tomita M, Wanner BL, Mori H (2006). Construction of *Escherichia coli* K-12 in-frame, single-gene knockout mutants: the Keio collection. Mol Syst Biol.

[CR2] Bartolome B, Jubete Y, Martinez E, de la Cruz F (1991). Construction and properties of a family of pACYC184-derived cloning vectors compatible with pBR322 and its derivatives. Gene.

[CR3] Benemann J (1996). Hydrogen biotechnology: progress and prospects. Nat Biotechnol.

[CR4] Blokesch M, Magalon A, Böck A (2001). Interplay between the specific chaperone-like proteins HybG and HypC in maturation of hydrogenases 1, 2, and 3 from *Escherichia coli*. J Bacteriol.

[CR5] Böck A, King PW, Blokesch M, Posewitz MC (2006). Maturation of hydrogenases. Adv Microb Physiol.

[CR6] Burgdorf T, Lenz O, Buhrke T, van der Linden E, Jones AK, Albracht SP, Friedrich B (2005). [NiFe]-hydrogenases of *Ralstonia eutropha* H16: modular enzymes for oxygen-tolerant biological hydrogen oxidation. J Mol Microbiol Biotechnol.

[CR7] Burgdorf T, van der Linden E, Bernhard M, Yin QY, Back JW, Hartog AF, Muijsers AO, de Koster CG, Albracht SP, Friedrich B (2005). The soluble NAD^+^-reducing [NiFe]-hydrogenase from *Ralstonia eutropha* H16 consists of six subunits and can be specifically activated by NADPH. J Bacteriol.

[CR8] Casadaban MJ, Cohen SN (1979). Lactose genes fused to exogenous promoters in one step using a Mu-lac bacteriophage: in vivo probe for transcriptional control sequences. Proc Nat Acad Sci USA.

[CR9] Deplanche K, Calderari I, Mikheenko IM, Sargent F, Macaskie LE (2010). Involvement of hydrogenases in the formation of highly catalytic Pd(0) nanoparticles by bioreduction of Pd(II) using *Escherichia coli* mutant strains. Microbiology.

[CR10] Drapal N, Böck A (1998). Interaction of the hydrogenase accessory protein HypC with HycE, the large subunit of *Escherichia coli* hydrogenase 3 during enzyme maturation. Biochemistry.

[CR11] Ghosh D, Bisaillon A, Hallenbeck PC (2013). Increasing the metabolic capacity of *Escherichia coli* for hydrogen production through heterologous expression of the *Ralstonia eutropha* SH operon. Biotechnol Biofuels.

[CR12] Hamilton CM, Aldea M, Washburn BK, Babitzke P, Kushner SR (1989). New method for generating deletions and gene replacements in *Escherichia coli*. J Bacteriol.

[CR13] Hartwig S, Thomas C, Krumova N, Quitzke V, Turkowsky D, Jehmlich N, Adrian L, Sawers RG (2015). Heterologous complementation studies in *Escherichia coli* with the Hyp accessory protein machinery from Chloroflexi provide insight into [NiFe]-Hydrogenase large subunit recognition by the HypC Protein Family. Microbiology.

[CR14] Jack RL, Buchanan G, Dubini A, Hatzixanthis K, Palmer T, Sargent F (2004). Coordinating assembly and export of complex bacterial proteins. EMBO J.

[CR15] Jacobi A, Rossmann R, Böck A (1992). The *hyp* operon gene products are required for the maturation of catalytically active hydrogenase isoenzymes in *Escherichia coli*. Arch Microbiol.

[CR16] Kelly CL, Pinske C, Murphy BJ, Parkin A, Armstrong F, Palmer T, Sargent F (2015). Integration of an [FeFe]-hydrogenase into the anaerobic metabolism of *Escherichia coli*. Biotechnol Rep.

[CR17] Lubitz W, Ogata H, Rudiger O, Reijerse E (2014). Hydrogenases. Chem Rev.

[CR18] Lyons LB, Zinder ND (1972). Genetic map of filamentous Bacteriophage F1. Virology.

[CR19] McDowall JS, Murphy BJ, Haumann M, Palmer T, Armstrong FA, Sargent F (2014). Bacterial formate hydrogenlyase complex. Proc Nat Acad Sci USA.

[CR200] Palmer T, Berks BC, Sargent F (2010). Analysis of Tat targeting function and twin-arginine signal peptide activity in Escherichia coli. Methods Mol Biol.

[CR20] Pinske C, Bonn M, Kruger S, Lindenstrauss U, Sawers RG (2011). Metabolic deficiencies revealed in the biotechnologically important model bacterium *Escherichia coli* BL21(DE3). PLoS ONE.

[CR21] Puigbo P, Guzman E, Romeu A, Garcia-Vallve S (2007). OPTIMIZER: a web server for optimizing the codon usage of DNA sequences. Nucleic Acids Res.

[CR22] Sambrook J, Russell DW (2001). Molecular cloning: a laboratory manual.

[CR23] Sargent F (2016). The model [NiFe]-Hydrogenases of *Escherichia coli*. Adv Microb Physiol.

[CR24] Schiffels J, Selmer T (2015). A flexible toolbox to study protein-assisted metalloenzyme assembly in vitro. Biotechnol Bioeng.

[CR25] Schiffels J, Pinkenburg O, Schelden M, Aboulnaga EHA, Baumann ME, Selmer T (2013). An innovative cloning platform enables large-scale production and maturation of an oxygen-tolerant [NiFe]-hydrogenase from *Cupriavidus necator* in *Escherichia coli*. PLoS ONE.

[CR26] Stripp ST, Lindenstrauss U, Sawers RG, Soboh B (2015). Identification of an isothiocyanate on the HypEF complex suggests a route for efficient cyanyl-group channeling during [NiFe]-Hydrogenase cofactor generation. PLoS ONE.

[CR27] Tabor S, Richardson CC (1985). A bacteriophage T7 RNA polymerase/promoter system for controlled exclusive expression of specific genes. Proc Nat Acad Sci USA.

[CR28] Wolf I, Buhrke T, Dernedde J, Pohlmann A, Friedrich B (1998). Duplication of *hyp* genes involved in maturation of [NiFe] hydrogenases in *Alcaligenes eutrophus* H16. Arch Microbiol.

